# Prolyl‐Hydroxyproline Promotes Structural Matrix Organization in 3D Equine Tendon Proper and Peritenon Post‐Injury Model

**DOI:** 10.1002/jor.70284

**Published:** 2026-09-21

**Authors:** Angelique O. Wong, Michael J. Mienaltowski

**Affiliations:** ^1^ Department of Animal Science, College of Agricultural & Environmental Sciences University of California Davis Davis California USA

**Keywords:** Equine tendon repair, extrinsic repair, matrix organization, peritenon, prolyl‐hydroxyproline

## Abstract

Tendinopathy of the superficial digital flexor tendon affects horse health and performance, with slow healing and high re‐injury rates due to the formation of mechanically weaker scar tissue. Tendons are rich in Type I collagen and may benefit from supplementation with collagen‐derived dipeptide prolyl hydroxyproline (H‐Pro‐Hyp‐OH) which improves collagen regeneration and reduces scarring in murine injury models. However, its effects on equine tenocytes in the tendon proper (TP) and peritenon (PERI) under stress are unknown. Three‐dimensional fibrin‐based TP and PERI constructs were used to evaluate the effects of H‐Pro‐Hyp‐OH within TP and PERI niches under control conditions and simulated post‐injury environments. Constructs were treated with H‐Pro‐Hyp‐OH alone or in combination with inflammatory (Interleukin‐1 beta, IL‐1β) or glucocorticoid (dexamethasone, DEX) stimuli. Gene expression was assessed by RT‐qPCR, collagen content was quantified using a hydroxyproline assay, and tendon ultrastructure was evaluated using second harmonic generation imaging. Under control conditions, H‐Pro‐Hyp‐OH enhanced alignment in TP. Under inflammatory and DEX stress, H‐Pro‐Hyp‐OH did not reverse stress‐induced transcriptional shifts or restore collagen content. However, H‐Pro‐Hyp‐OH treatment improved collagen fibril organization and alignment, with structural rescue seen in PERI constructs. In stressful conditions induced by IL‐1β or DEX, structural improvements did not always correspond with biochemical measurements of collagen, suggesting that H‐Pro‐Hyp‐OH promotes matrix organization rather than bulk collagen accumulation in adverse post‐injury environments. The responsiveness of PERI cells highlights the importance of targeting the extrinsic repair niche to promote functional tendon repair and limit fibrotic scarring.

## 1 Introduction

The superficial digital flexor tendon (SDFT) is a high‐strain, principal energy‐storing tendon in the horse that serves as a functional equivalent to the human Achilles tendon [1, 2, 3, 4, 5, 6, 7, 8, 9]. A majority of tendinopathies in equine athletes occur in the SDFT [6, 10]. In adult tendinopathies, the healing process is slow and tendon lesions are typically replaced by disorganized, structurally deficient fibrous scar tissue. The scar tissue is mechanically weaker than uninjured native tissue, leading to high re‐injury rates that impact the long‐term health and performance of equine athletes [5, 6, 8, 10, 11, 12, 13].

The hierarchical collagenous matrix of the SDFT is synthesized and maintained by two transcriptionally and regionally distinct cellular niches: the tendon proper (TP) and the peritenon (PERI) [12, 14, 15, 16, 17, 18, 19, 20]. While PERI‐derived cells exhibit greater plasticity and serve as highly responsive mediators of repair, they are less likely to produce mature, type I collagen‐rich tissue [13, 16, 20, 21]. Fibroblasts that initially infiltrate a tendon lesion to synthesize a provisional matrix frequently originate from the paratenon, highlighting PERI as a crucial source of extrinsic repair cells [13, 16, 20, 21]. Additionally, PERI cells exert trophic effects on TP cells via paracrine signaling, secreting growth factors that stimulate tenogenic differentiation [20, 21]. Conversely, while TP cells possess greater tenogenic capacity, they remain less active during the initial injury response and are more prone to depositing calcified, scar‐like matrix under osteogenic conditions [4, 19, 20, 21]. The mechanisms underlying differential responses of TP and PERI cells during tendon healing are not yet fully understood, especially in mature tendon [13, 21]. While neonatal tendon lesions can regenerate native functional tissue, adult tendon often heals through fibrotic scar tissue deposition [8, 10, 11, 13]. Thus, considering cell populations from each niche is crucial to understanding tendon repair.

Following injury, the local microenvironment is further compromised by inflammation and common clinical interventions. Tenocytes and immune cells release pro‐inflammatory cytokines, such as IL‐1β, which downregulate type I collagen expression (*COL1A1*) and drive tenocytes toward an activated inflammatory and fibrotic phenotype [22, 23, 24, 25, 26, 27]. Il‐1β also suppresses key tenogenic transcription factors such as Scleraxis (*SCX*) and inhibits matrix assembly genes [25, 26]. While glucocorticoids, such as dexamethasone (DEX), are widely used to clinically manage inflammation in tendinopathies, long‐term steroid exposure can have paradoxical and detrimental effects on repair. By inducing a catabolic shift that suppresses tenogenic pathways and collagen synthesis, DEX treatment may compromise the mechanical integrity of the ECM, potentially increasing the risk of tendon rupture [28, 29, 30, 31, 32].

Collagen‐derived dipeptide prolyl hydroxyproline (H‐Pro‐Hyp‐OH) functions as a signaling molecule to promote fibroblast proliferation and matrix remodeling, and it also serves as a resource for collagen synthesis [33, 34, 35, 36, 37]. In mouse and human Achilles tenocytes, H‐Pro‐Hyp‐OH has been shown to promote organized collagen synthesis, fibroblast proliferation, and matrix remodeling [33, 34, 35, 36]. Understanding how both TP and PERI niches respond to H‐Pro‐Hyp‐OH supplementation on TP and PERI tenocytes under normal and simulated injury conditions may reveal injury response mechanisms that favor organized tenogenesis rather than disorganized fibrotic repair. We hypothesized that H‐Pro‐Hyp‐OH supplementation would promote tenogenic differentiation and matrix assembly in engineered equine tendon constructs, even when stimulated with clinically relevant stressors, inflammation (IL‐1β) or glucocorticoid (DEX) treatment, by bolstering collagen synthesis, fibril organization, and tissue remodeling. An initial study was conducted to establish the optimal tenogenic concentration of H‐Pro‐Hyp‐OH under control conditions. The optimal dose was subsequently applied concurrently with either an inflammatory or glucocorticoid stimulus to evaluate the dipeptide's tenogenic effects.

## 2 Methods

### 2.1 2D Culture

Tendon proper (TP) and peritenon (PERI) tenocytes were previously isolated from the SDFT of six horses (Quarter Horses, Thoroughbreds, and one Appendix horse; ages 4–12) with no known history of tendinopathy [15, 16]. Subjects were euthanized for reasons unrelated to this study and were thus exempt from approval by the University of California Davis Institutional Animal Care and Use Committee. Cells were isolated from a 2.5 cm‐long segment of SDFT harvested approximately 10 to 15 cm proximal to the forelimb fetlock. Cells from the inner 2–2.5 mm of the central tendon (TP) and the outer 1 mm of the epitenon and the paratenon (collectively, PERI) were isolated using enzymatic digestion of the tissue. After two passages, isolated primary cells were cryopreserved in 10% dimethyl sulfoxide in complete media containing 10% fetal bovine serum, 1% antibiotic‐antimycotic, and 1% l‐glutamine, and then stored in liquid nitrogen until their use. For this study, the cryopreserved second passage TP and PERI tenocytes were thawed and expanded in α‐MEM complete media, which was replaced every 48 h. Upon reaching 85% confluency, cells were rinsed in Dulbecco's Phosphate Buffered Saline and trypsinized using 0.25% Trypsin EDTA for fibrin scaffold seeding.

### 2.2 3D Culture

TP and PERI tenocytes were each seeded at 3 × 10^5^ cells/construct in a fibrin‐based hydrogel, which acts as a provisional matrix for the cells [38]. The gel was placed on a silicone elastomer substrate between two brushite cement anchors, pinned 10 mm apart in a 6‐well culture plate. As the fibrin gel contracts around the anchors, the construct generates uniaxial longitudinal tension on the cells, analogous to mechanical cues found during in vivo development and early repair in the developing SDFT [38].

To determine the effect of H‐Pro‐Hyp‐OH in promoting tenogenesis in TP and PERI constructs, we first provided constructs with 0, 200, or 500 µg/mL H‐Pro‐Hyp‐OH (catalog #4001630 from Bachem Americas Inc; or catalog #A670815 from Ambeed Inc) under control conditions in complete media with 200 μM ascorbic‐2‐phosphate replaced every 48 h. These concentrations were previously shown to be highly effective at promoting proliferation, increased *Scx* and *Mkx* expression, and increased amounts of collagen in primary cell culture of mouse cells [36].

To evaluate whether H‐Pro‑Hyp‐OH can counteract IL‑1β‑ and dexamethasone‑induced limitations in tendon proper and peritenon cell phenotype and matrix production, constructs were grown for 5 days in complete media with 200 μM ascorbic‐2‐phosphate, then stimulus groups were stressed with either IL‐1β or DEX, while stimulus+treatment groups were stressed and concurrently treated with 500 µg/mL H‐Pro‐Hyp‐OH. To simulate detrimental post‐injury inflammation, constructs received 500 µg/mL H‐Pro‐Hyp‐OH and 5 ng/mL recombinant equine interleukin 1 beta (IL‐1β) (R&D Systems), an inflammatory cytokine typically found in tendon lesions, in complete media with 200 μM ascorbic‐2‐phosphate [25]. TP and PERI constructs were treated with 500 µg/mL H‐Pro‐Hyp‐OH and 10 nM dexamethasone (DEX) (Sigma‐Aldrich) in complete media with 200 μM ascorbic‐2‐phosphate to determine their effects in the same niche, especially since DEX has paradoxical effects on injured tendons [23].

All constructs received 200 µM ascorbic‐2‐phosphate, given its tenogenic properties [12, 15, 18, 39]. Tendon constructs were collected on the 11th day of growth and rinsed in phosphate‐buffered saline to remove dipeptide and media residue before being snap‐frozen in liquid nitrogen for downstream gene expression analyses and collagen content assays, or fixed in 10% neutral buffered formalin and stored in 70% ethanol for quantitative ultrastructural imaging.

### 2.3 RNA Isolation and RT‐qPCR

Snap‐frozen tendon constructs were homogenized in TRIzol reagent (Thermo Fisher Scientific) using a Tissue‐Tearor (Biospec Products). After phase separation with chloroform, the upper aqueous phase was applied to the Qiagen RNeasy Plus Micro Kit following the kit instructions with on‐column DNase treatment). Total RNA concentration was measured, and purity was assessed for each sample using a NanoDrop spectrophotometer (Thermo Fisher Scientific).

Reverse transcription was performed using a High‐Capacity cDNA Reverse Transcription Kit (Applied Biosystems) with 380 ng of total RNA per reaction. Each quantitative polymerase chain reaction (qPCR) was performed with a cDNA template from the equivalent of 10 ng of total RNA for each sample. The template cDNA was combined with TaqMan™ Fast Advanced Master Mix for qPCR and TaqMan™ gene assays to a final volume of 20 µL, assessed in duplicate. Relative gene expression was quantified for markers of tenogenic differentiation (*SCX*, *MKX*), ECM assembly (*COL1A1, DCN, LOX*), perivascular cell differentiation (*CSPG4*), and apoptosis (*CASP3*), normalized to *POLR2A* [15]. *COL1A1* was chosen as the primary structural marker because Type I collagen comprises the majority of organized collagenous ECM in tendon [12, 14, 15, 16, 17, 18]. Relative gene expression was calculated using the relative quantity ratio formula based on the C_T_ and the amplification efficiency, which was calculated using LinRegPCR v7.0 [40, 41].

### 2.4 Hydroxyproline Assay

A hydroxyproline assay was performed according to a modified previously reported protocol to quantify collagen content [42, 43]. Briefly, tendon constructs were removed and dried at 120°C to record dry mass. To hydrolyze soluble proteins, samples were submerged in 200 μL 6 M HCl and incubated in a heating block at 120°C for 2 h. Following hydrolysis, samples were briefly centrifuged and incubated uncapped at 120°C for 1.5 h to evaporate HCl. The resulting pellets were resuspended in 200 μL hydroxyproline buffer (0.173 M citric acid, 0.140 M acetic acid, 0.570 M NaOH in 500 mL total volume water, pH 6–6.5), then diluted using a 1:5 sample to buffer ratio. 150 μL of Chloramine‐T (14.1 mg/mL) is added to each sample and incubated for 20 min to act as the oxidizing agent. 150 μL aldehyde‐perchlorate solution (8.02 M 1‐propanol, 3.03 M 70% perchloric acid,1 M 4‐dimethylamino benzaldehyde in 10 mL total volume by water) is added as the color‐forming agent. Samples are incubated at 60°C for 15 min to accelerate chromophore production. Hydroxyproline concentration was determined by measuring absorbance at 550 nm, and absorbance values were converted to micrograms of hydroxyproline by standard curve comparison. Total collagen percent was calculated based on the assumption that the mammalian Achilles tendon contains 13.7% hydroxyproline in collagen.

### 2.5 Second Harmonic Generation Microscopy

#### 2.5.1 Imaging

Tendon ultrastructure was visualized using second harmonic generation (SHG) imaging on a Leica TCS SP8 MP upright multiphoton confocal microscope (Leica Microsystems GmbH, Wetzlar, Germany) using a Mai Tai DeepSee laser (Spectra‐Physics, Milpitas, CA) tunable from 690 to 1090 nm, set to an excitation wavelength of 866 nm. Z‐stacks were acquired with a step size of z = 0.85 μm, using a 25X water‐immersion objective (Leica HC FLUOTAR L 25x/0.95 W VISIR). Constructs were submerged in 50% glycerol mounting medium, with petroleum jelly used as an occlusive to seal the cover slip and steel washer spacers during imaging.

#### 2.5.2 Analysis

SHG images were processed and analyzed using Fiji (ImageJ, NIH) with the OrientationJ v 2.0.7 plugin (Biomedical Imaging Group, EPFL, Switzerland) to assess collagen content and collagen fiber organization [44, 45]. 3D images were compressed into 2D using maximum intensity Z projection; representative images can be seen in the Supplemental Materials Fig. S1 (A–F) and Fig. S2 (A–H). To assess local collagen fibril orientation and directionality, OrientationJ Analysis was used to generate energy, coherence, and orientation maps based on calculating the local gradient structure tensor using Gaussian smoothing, with a tensor radius of 1.0 and a gradient window of 4 pixels. To delineate collagen signals, the energy map was thresholded and converted to a binary mask. Fibrillar collagen content was quantified as the signal area fraction of the preprocessed energy map. The final analysis map was generated by multiplying the coherence/orientation map and the binarized energy mask using Image Calculator to remove background noise. Anisotropy was measured as the mean gray value of a binary masked coherence map for local fiber coherence. OrientationJ Distribution was then applied to the masked orientation map to calculate the overall angular distribution of collagen fibers. A Gaussian curve was fitted to the resulting histogram using the Curve Fitting tool, where the dominant fiber orientation was defined as the peak of the histogram (c), and the standard deviation (d) was reported as the angular dispersion or spread of collagen fibers. Standard deviation was used to calculate the Full Width at Half Maximum (FWHM) of the Gaussian fit to quantify fiber organization [46].

$$y=a+\left(b-a\right)\;\text{exp}\left(-\frac{{\left(x-c\right)}^{2}}{2d^{2}}\right)$$

### 2.6 Statistics

Statistical analysis was performed using a repeated measures mixed‐effects model to evaluate main effects (concentration, cell type, interaction), followed by post‐hoc multiple comparison Sidak tests for select planned comparisons (groups within cell type and across cell type) in GraphPad Prism v. 10 (Graphpad Software, INC., San Diego, CA). Expression data were normally distributed upon log transformation; hydroxyproline assay and SHG imaging data were applied directly to mixed‐effects model analysis. Before application to mixed‐effects model analysis, outliers were removed using Grubbs' outlier test. Statistical significance was defined as (* = *p* ≤ 0.05 and ** = *p* < 0.01) while trends were reported as (*p* < 0.10).

## 3 Results

### 3.1 Effects of H‐Pro‐Hyp‐OH Alone

**Figure 1:**
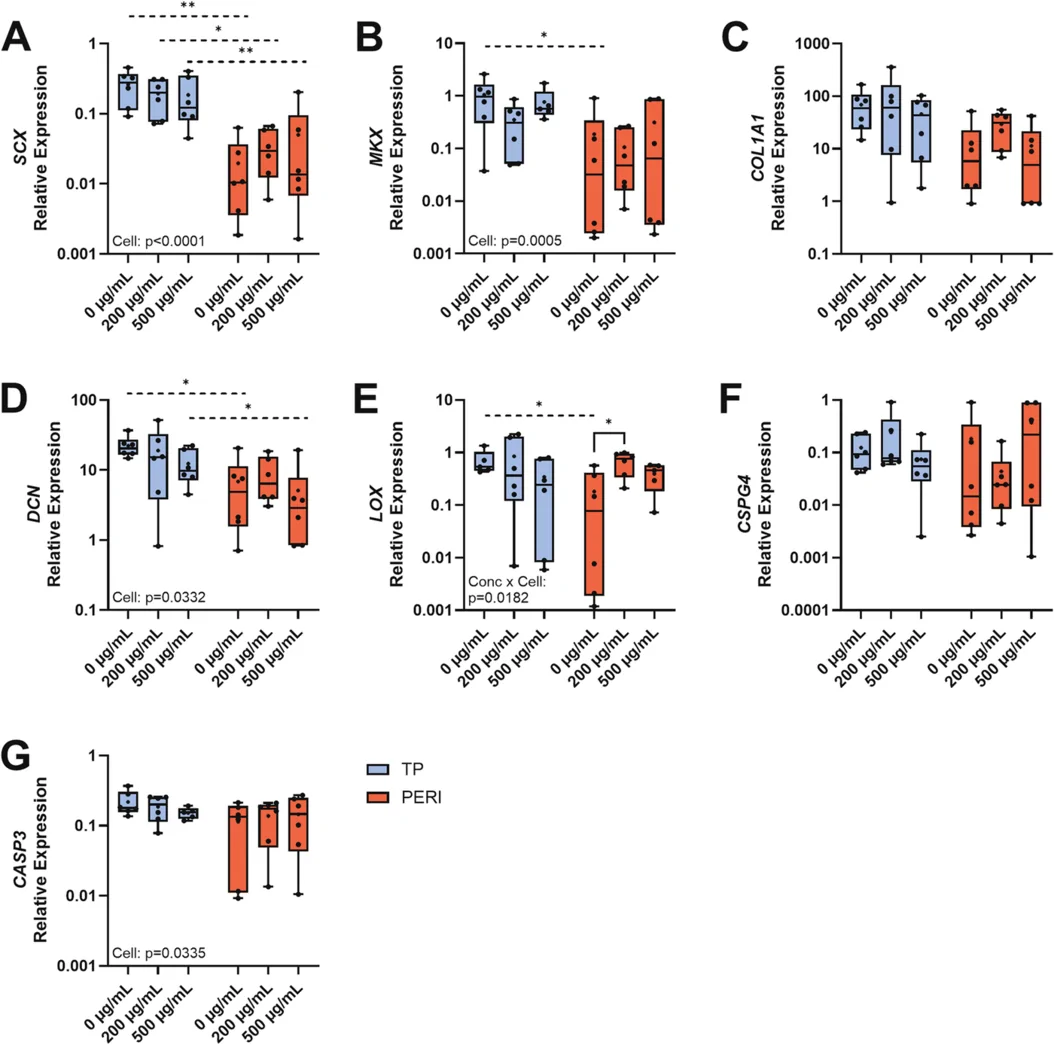
Gene expression profiles of TP and PERI constructs treated with H‐Pro‐Hyp‐OH under control conditions. RT‐qPCR was performed to measure mRNA expression levels for *SCX* (A), *MKX* (B). *COL1A1* (C), *DCN* (D). *LOX* (E), *CSPG4* (F), and *CASP3* (G) in TP and PERI constructs following treatment with 0, 200, and 500 μg/mL H‐Pro‐Hyp‐OH. Data are presented as log relative expression with box plots with mean as “+” and individual points plotted; data are normalized to *POLR2A*. Statistical analysis was performed using repeated measures mixed‐effects model with Sidak tests used for post‐hoc multiple comparisons; outliers were removed using Grubbs' outlier test. Statistical significance was defined as (* = *p* ≤ 0.05 and ** = *p* < 0.01; *n* = 5–6).

**Figure 2:**
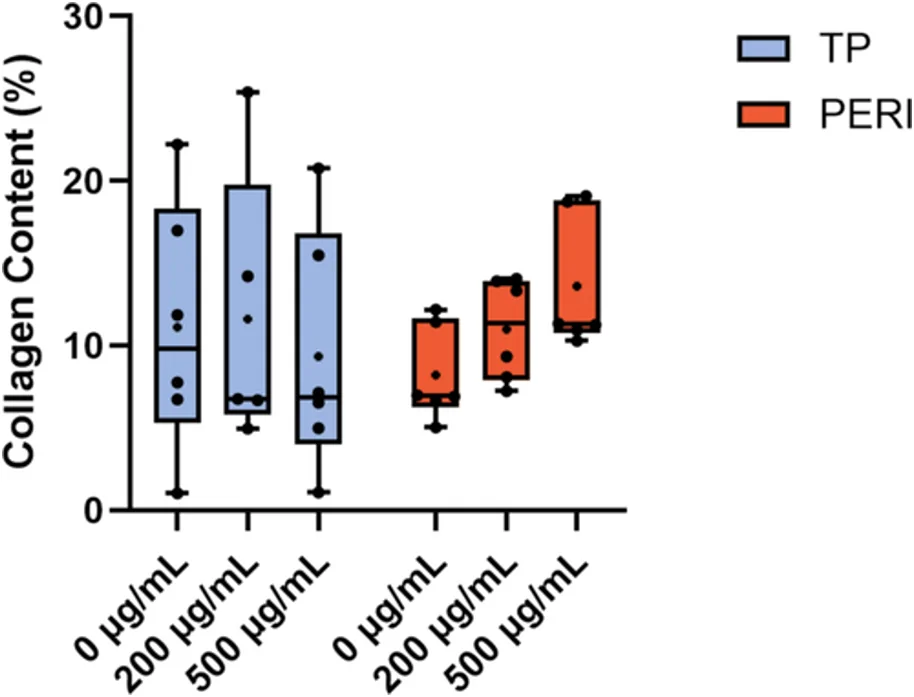
Total collagen content of TP and PERI constructs treated with H‐Pro‐Hyp‐OH. Total collagen content was determined using a modified hydroxyproline assay for TP and PERI constructs treated with 0, 200, and 500 μg/mL H‐Pro‐Hyp‐OH. Data are presented as percent collagen content and displayed as box plots with mean as “+” and individual points plotted. Statistical analysis was performed using repeated measures mixed‐effects model with Sidak tests used for post‐hoc multiple comparisons; outliers were removed using Grubbs' outlier test. Statistical significance was defined as (* = *p* ≤ 0.05 and ** = *p* < 0.01; *n* = 5–6).

**Figure 3:**
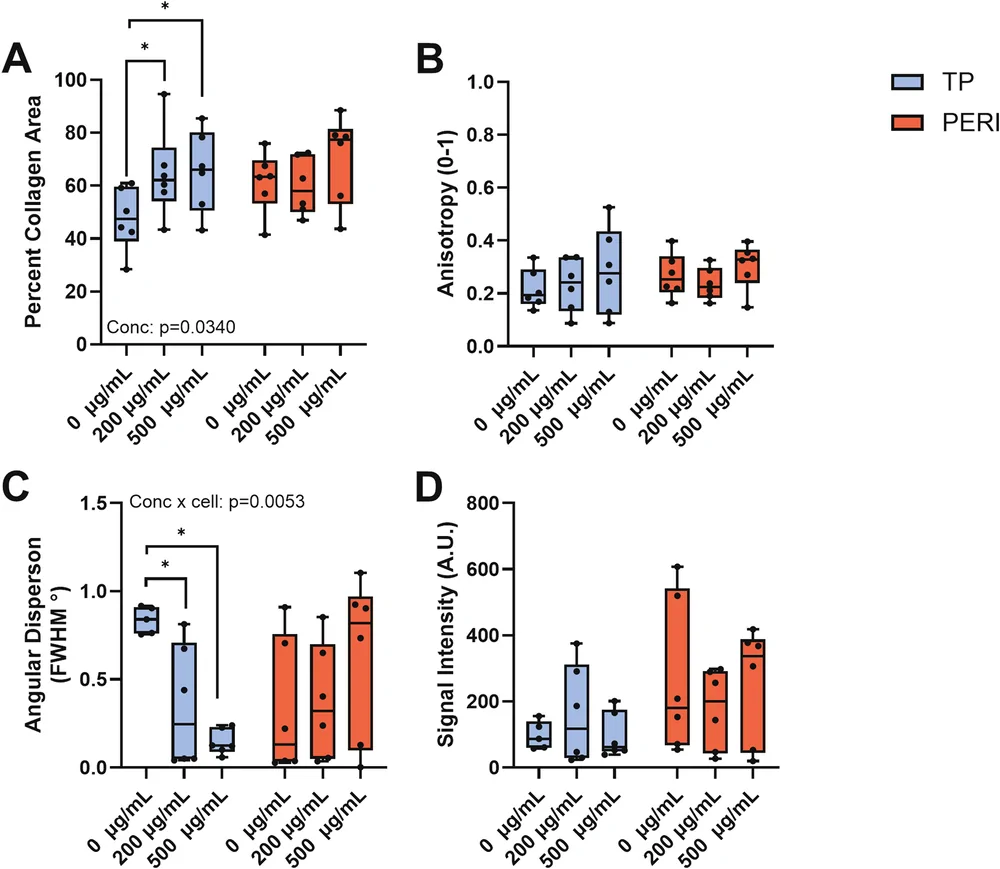
Quantitative SHG image analysis of TP and PERI constructs treated with H‐Pro‐Hyp‐OH under control conditions. SHG image analysis of TP and PERI constructs following treatment with 0, 200, or 500 μg/mL H‐Pro‐Hyp‐OH. Percent collagen area (A) represents total fibrillar collagen quantified as the SHG signal area fraction of a binarized energy map. Anisotropy (B) represents local fiber coherence on a 0–1 scale, where 1 is perfect linearity/alignment. Angular dispersion (FWHM) (C) represents the overall fiber alignment spread, derived from a Gaussian fit of the angle orientation distribution. SHG signal intensity (D) represents fiber packing density. Data are expressed as box plots with mean as “+” and individual points plotted. Statistical analysis was performed using repeated measures mixed‐effects model with Sidak tests used for post‐hoc multiple comparisons; outliers were removed using Grubbs' outlier test. Statistical significance was defined as (* = *p* ≤ 0.05 and ** = *p* < 0.01; *n* = 5–6).

Under control conditions, H‐Pro‐Hyp‐OH supplementation resulted in cell‐type‐dependent effects on mRNA expression, collagen content, and organization. *SCX* and *DCN* expression levels were generally greater in TP constructs than PERI constructs (Figure 1A,D); MKX and LOX expression levels were also greater in control TP constructs relative to PERI constructs (Figure 1B,E). *LOX* expression in PERI constructs treated with 200 ug/mL H‐Pro‐Hyp‐OH were higher when compared to the control (Figure 1E). Expression differences were not seen for *COL1A1*, *CSPG4*, and *CASP3* (Figure 1C,F,G). In PERI constructs, total percent collagen measured by hydroxyproline assay had an increasing trend at 500 μg/mL H‐Pro‐Hyp‐OH (*p *= 0.0651), while collagen content remained statistically unchanged across all dipeptide concentrations under control conditions in TP constructs (Figure 2). Conversely, the percent collagen area measured by SHG in TP constructs was significantly increased at 200 and 500 μg/mL H‐Pro‐Hyp‐OH (Figure 3A), while angular dispersion was greatly reduced at 500 μg/mL (Figure 3C). No significant changes were seen for anisotropy (Figure 3B) and signal intensity (Figure 3D).

### 3.2 IL‐1β Challenge

**Figure 4:**
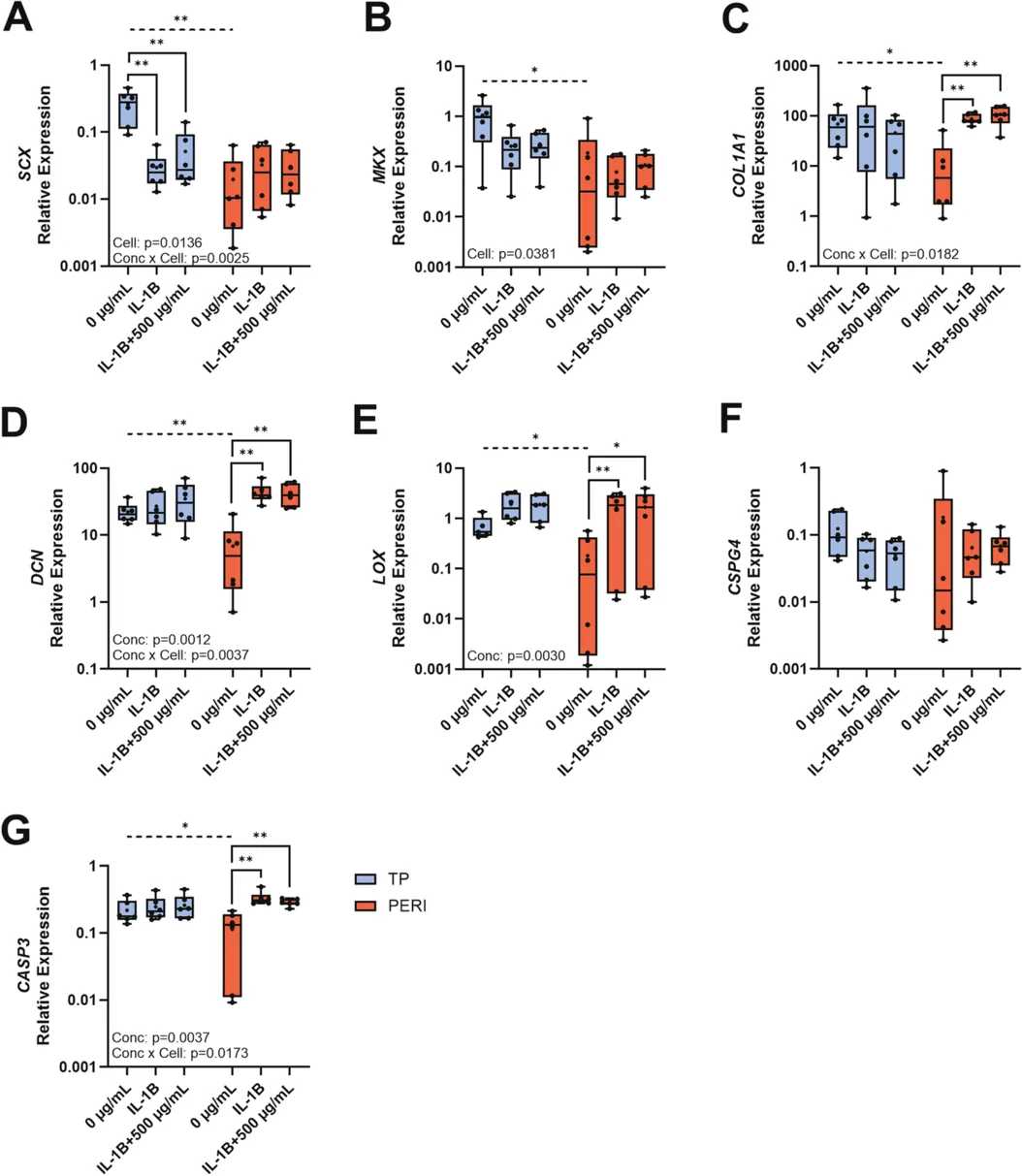
Gene expression profiles of TP and PERI constructs under IL‐1β induced inflammatory stress treated with H‐Pro‐Hyp‐OH. RT‐qPCR was performed to measure mRNA expression levels of *SCX* (A), *MKX* (B). *COL1A1* (C), *DCN* (D). *LOX* (E),*CSPG4* (F), and *CASP3* (G) in TP and PERI constructs following treatment with 5 ng/mL IL‐1β and 0 or 500 μg/mL H‐Pro‐Hyp‐OH. Data are presented as log relative expression with box plots with mean as “+” and individual points plotted; data are normalized to *POLR2A*. Statistical analysis was performed using repeated measures mixed‐effects model with Sidak tests used for post‐hoc multiple comparisons; outliers were removed using Grubbs' outlier test. Statistical significance was defined as (* = *p* ≤ 0.05 and ** = *p* < 0.01; *n* = 5–6).

**Figure 5:**
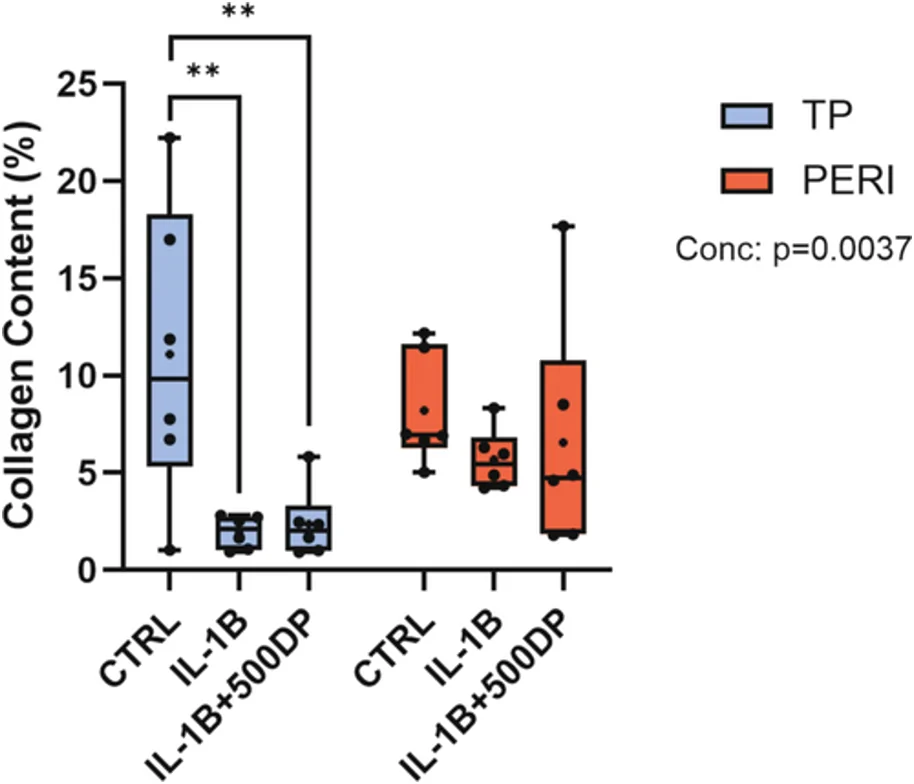
Total collagen content of TP and PERI constructs under inflammatory stress treated with H‐Pro‐Hyp‐OH. Total collagen content was determined for TP and PERI constructs using a modified hydroxyproline assay following treatment with 5 ng/mL IL‐1β and 0 or 500 μg/mL H‐Pro‐Hyp‐OH. Data are presented as percent collagen content and displayed as box plots with mean as “+” and individual points plotted. Statistical analysis was performed using repeated measures mixed‐effects model with Sidak tests used for post‐hoc multiple comparisons; outliers were removed using Grubbs' outlier test. Statistical significance was defined as (* = *p* ≤ 0.05 and ** = *p* < 0.01; *n* = 6).

**Figure 6:**
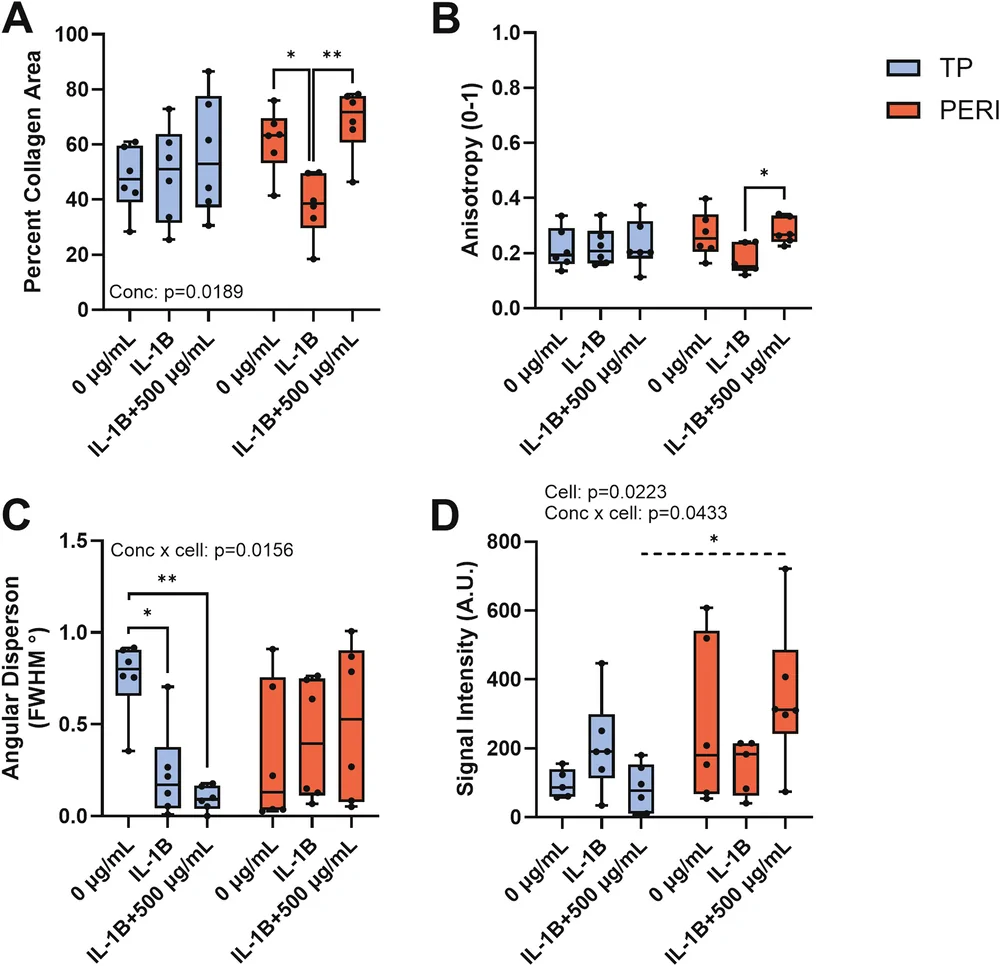
Quantitative SHG image analysis of TP and PERI constructs under IL‐1β induced inflammatory stress treated with H‐Pro‐Hyp‐OH. SHG image analysis of TP and PERI constructs following treatment with 5 ng/mL IL‐1β and 0 or 500 μg/mL H‐Pro‐Hyp‐OH. Percent collagen area (A) represents total fibrillar collagen quantified as the SHG signal area fraction of a binarized energy map. Anisotropy (B) represents local fiber coherence on a 0–1 scale, where 1 is perfect linearity/alignment. Angular dispersion (FWHM) (C) represents the overall fiber alignment spread, derived from a Gaussian fit of the angle orientation distribution. SHG signal intensity (D) represents fiber packing density. Data are expressed as box plots with mean as “+” and individual points plotted. Statistical analysis was performed using repeated measures mixed‐effects model with Sidak tests used for post‐hoc multiple comparisons; outliers were removed using Grubbs' outlier test. Statistical significance was defined as (* = *p* ≤ 0.05 and ** = *p* < 0.01; *n* = 5–6).

IL‐1β stimulation in TP and PERI constructs induced a distinct inflammatory response that was partially rescued by H‐Pro‐Hyp‐OH treatment. Generally, tenogenic markers SCX and MKX had greater expression in TP constructs (Figure 4A,B). Moreover, tenogenic marker *SCX* was significantly suppressed in TP constructs, and 500 μg/mL H‐Pro‐Hyp‐OH supplementation did not successfully restore this downregulation (Figure 4A). In contrast, inflammatory stress in PERI constructs resulted in a significant upregulation of *COL1A1*, *LOX*, and *DCN* in both IL‐1β and IL‐1β + 500 μg/mL H‐Pro‐Hyp‐OH groups (Figure 4C–E) along with an increase in apoptosis marker *CASP3* following IL‐1β stimulus (Figure 4G). Expression differences were not seen for *CSPG4* (Figure 4F). Total collagen content quantified via hydroxyproline assay revealed that IL‐1β treatment resulted in a decrease in total percent collagen in TP constructs, which persisted despite H‐Pro‐Hyp‐OH supplementation (Figure 5). Morphometric analysis using SHG imaging revealed a decrease in angular dispersion in TP constructs in both IL‐1β and IL‐1β + 500 μg/mL H‐Pro‐Hyp‐OH (+ 500) groups (Figure 6C). In PERI constructs, inflammatory stimulus resulted in a significant reduction in percent collagen area, which was successfully restored with dipeptide treatment (Figure 6A). Additionally, anisotropy in PERI constructs significantly increased in IL‐1β + 500 compared to IL‐1β only, indicating a successful rescue of collagen organization under inflammatory stress (Figure 6B). Signal intensity only differed when comparing across cell type for IL‐1β + 500 groups (Figure 6D).

### 3.3 DEX Challenge

**Figure 7:**
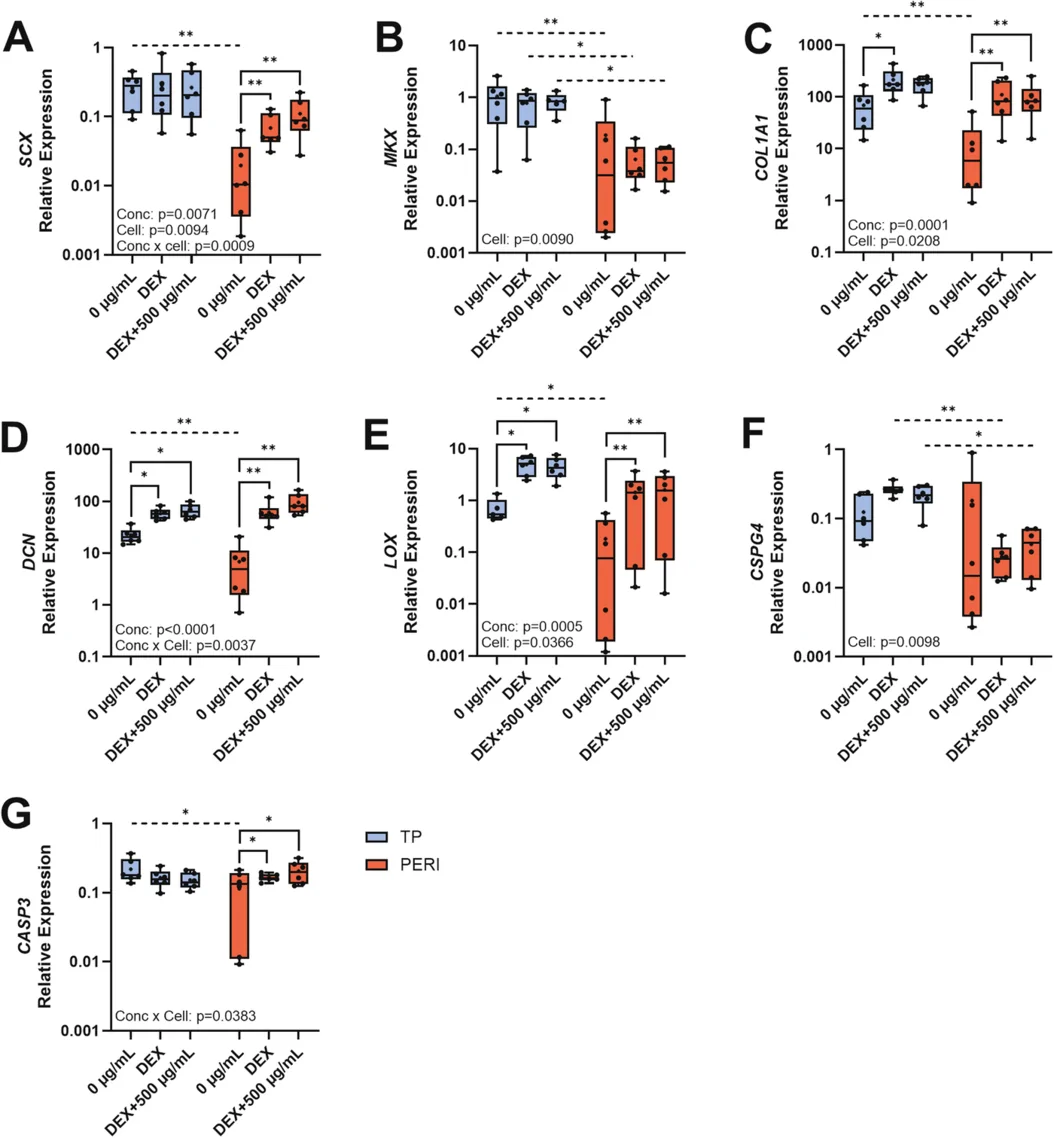
Gene expression profiles of TP and PERI constructs under DEX‐induced gluocorticoid stress treated with H‐Pro‐Hyp‐OH. RT‐qPCR was performed to measure mRNA expression levels for *SCX* (A), *MKX* (B). *COL1A1* (C), *DCN* (D). *LOX* (E), *CSPG4* (F), and *CASP3* (G) in TP and PERI constructs following treatment with 10 nM DEX and 0 or 500 μg/mL H‐Pro‐Hyp‐OH. Data are presented as log relative expression with box plots with mean as “+” and individual points plotted; data are normalized to *POLR2A*. Statistical analysis was performed using repeated measures mixed‐effects model with Sidak tests used for post‐hoc multiple comparisons; outliers were removed using Grubbs' outlier test. Statistical significance was defined as (* = *p* ≤ 0.05 and ** = *p* < 0.01; *n* = 5–6).

**Figure 8:**
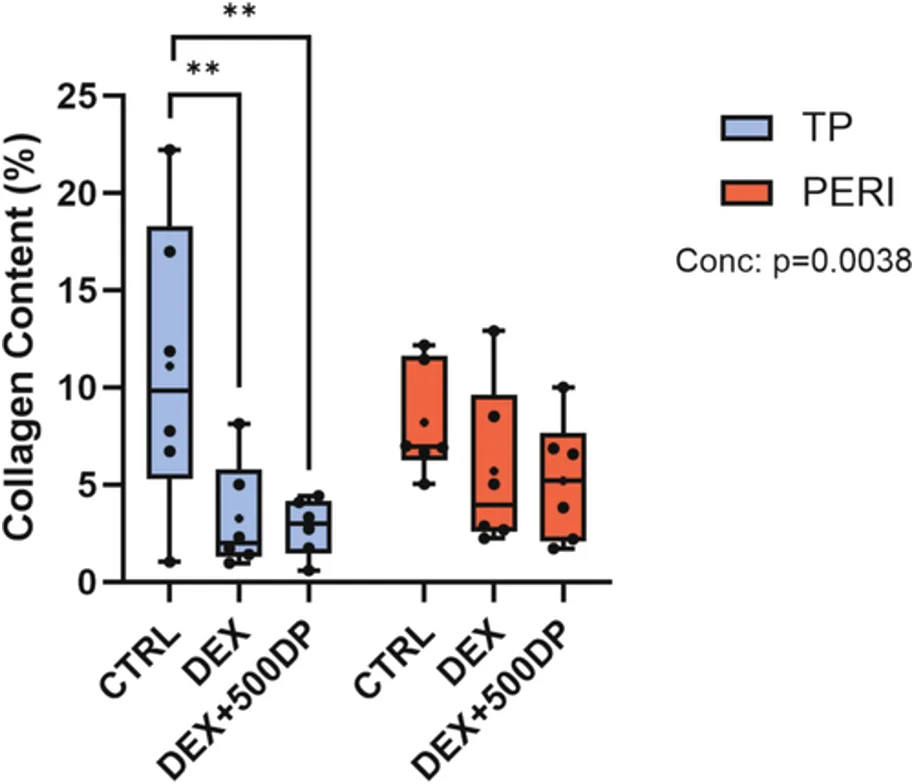
Total collagen content of TP and PERI constructs under DEX‐induced catabolic stress treated with H‐Pro‐Hyp‐OH. Total collagen content was determined using a modified hydroxyproline assay for TP and PERI constructs following treatment with 10 nM DEX and 0 or 500 μg/mL H‐Pro‐Hyp‐OH. Data are presented as percent collagen content and displayed as box plots with mean as “+” and individual points plotted. Statistical analysis was performed using repeated measures mixed‐effects model with Sidak tests used for post‐hoc multiple comparisons; outliers were removed using Grubbs' outlier test. Statistical significance was defined as (* = *p* ≤ 0.05 and ** = *p* < 0.01; *n* = 6).

**Figure 9:**
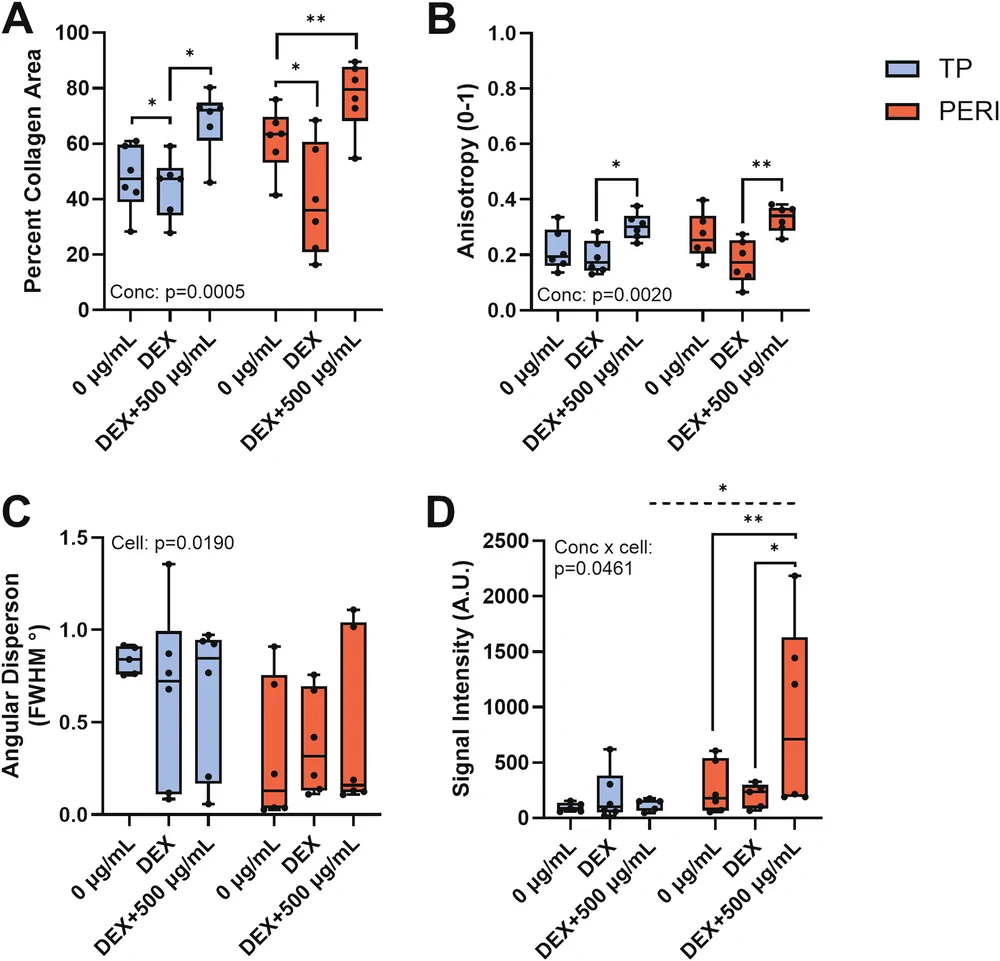
Quantitative SHG image analysis of TP and PERI constructs under DEX‐induced stress treated with H‐Pro‐Hyp‐OH. SHG image analysis of TP (A‐D) and PERI (E‐H) constructs following treatment with 10 nM DEX and 0 or 500 μg/mL H‐Pro‐Hyp‐OH. Percent collagen area (A) represents total fibrillar collagen quantified as the SHG signal area fraction of a binarized energy map. Anisotropy (B) represents local fiber coherence on a 0–1 scale, where 1 is perfect linearity/alignment. Angular dispersion (FWHM) (C) represents the overall fiber alignment spread, derived from a Gaussian fit of the angle orientation distribution. SHG signal intensity (D) represents fiber packing density. Data are expressed as box plots with mean as “+” and individual points plotted. Statistical analysis was performed using repeated measures mixed‐effects model with Sidak tests used for post‐hoc multiple comparisons; outliers were removed using Grubbs' outlier test. Statistical significance was defined as (* = *p* ≤ 0.05 and ** = *p* < 0.01; *n* = 5–6).

Tendon constructs responded to dexamethasone stress with an upregulation of matrix structure and regulatory genes. Both TP and PERI constructs demonstrated significantly higher expression of *COL1A1*, *LOX*, and *DCN* following DEX challenge compared to the control (Figure 7C–E). Expression remained high with dipeptide treatment but was not statistically different from DEX groups. In TP constructs, DEX treatment increased *CSPG4* expression levels in TP constructs compared to PERI constructs (Figure 7F). While *SCX* expression was lower in control PERI constructs relative to TP constructs, *SCX* expression in PERI constructs was significantly higher under DEX stress and remained elevated after H‐Pro‐Hyp‐OH treatment (Figure 7A). Cell‐type‐specific expression of *MKX* was seen with increased levels found in TP cell‐derived constructs over PERI cell‐derived constructs (Figure 7B). Expression of *CASP3* was also elevated after DEX treatment and also remained elevated in the midst of H‐Pro‐Hyp‐OH treatment (Figure 7G). Total percent collagen via hydroxyproline assay was lower in DEX and DEX + 500 TP constructs compared to the control (Figure 8). SHG imaging revealed a partial structural recovery mediated by H‐Pro‐Hyp‐OH in both cell populations. In TP and PERI constructs under dexamethasone challenge, dipeptide treatment resulted in a significant increase in percent collagen area (Figure 9A) and anisotropy (Figure 9B) compared to DEX. For PERI constructs, the addition of dipeptide treatment also increased signal intensity (Figure 9D). While cell‐type‐based differences in angular dispersion were seen between TP and PERI constructs, no specific comparison was significant in post‐hoc comparisons (Figure 9C).

## 4 Discussion

This study evaluated the efficacy of prolyl‐hydroxyproline (H‐Pro‐Hyp‐OH) to promote tenogenic markers in 3D equine SDFT TP and PERI models in a simulated post‐injury environment with inflammatory or glucocorticoid stress. Our findings demonstrate that PERI cells exhibit high repair plasticity and that H‐Pro‐Hyp‐OH supplementation facilitates organized fibrillogenesis. H‐Pro‐Hyp‐OH treatment generates a cell‐type specific response, primarily eliciting a regenerative and structural rescue response in PERI constructs, with targeted effects on collagen organization in TP constructs. Interestingly, mass percent collagen measured by hydroxyproline assay did not always parallel percent collagen area captured by SHG imaging, suggesting H‐Pro‐Hyp‐OH prioritizes matrix assembly over bulk protein accumulation. The hydroxyproline assay quantifies total collagen content, even collagen not used in a structure, while SHG imaging selectively visualizes the non‐centrosymmetric, crystalline structure of organized fibrillar collagen [18, 43, 47].

Dipeptide‐treated PERI constructs in control conditions exhibited a dose‐dependent increase in mass collagen content, highlighting the metabolic responsiveness of PERI cells and consistent with findings that H‐Pro‐Hyp‐OH acts as both a bioactive signaling molecule and as a building block/substrate resource for collagen synthesis [17, 33, 34, 35, 36]. In control PERI constructs, 500 μg/mL H‐Pro‐Hyp‐OH supplementation resulted in a dose‐dependent increase in total collagen content despite no corresponding increase in *COL1A1* gene expression. This mismatch suggests that in a homeostatic state, supplemented H‐Pro‐Hyp‐OH is utilized as an effective resource for collagen synthesis by increasing the supply of proline and hydroxyproline, enabling tenocytes to enhance matrix remodeling and collagen turnover without activating a transcriptional repair response that could result in aberrant expression [17, 33].

In comparison, the more concentrated response in TP constructs is consistent with tendon proper cells' specialized role in maintaining tendon ultrastructure rather than rapid matrix deposition. In TP constructs, the decrease in *DCN* expression and increase in SHG collagen area suggest a dipeptide‐mediated shift toward matrix organization and fibril alignment. Downregulation of *DCN* is more likely facilitating fibril alignment rather than relaxing the regulation of fibril spacing to allow for increased fibril aggregation, particularly given the lack of a statistically significant increase in SHG intensity [48]. Furthermore, the decrease in angular dispersion at 500 μg/mL H‐Pro‐Hyp‐OH supports the shift toward a more linear, organized alignment of collagen fibers. Taken together, this supports a model in which TP cells preferentially respond to H‑Pro‑Hyp‑OH by refining existing fibrillar architecture rather than increasing fibril diameter or packing density, thus reinforcing ultrastructural integrity without substantially altering bulk collagen content.

500 μg/mL H‐Pro‐Hyp‐OH was identified as an acceptable concentration to evaluate in post‐injury tendon models, as it elicited improvements in matrix organization in TP and bulk quantitative collagen in PERI cell populations. These results are consistent with the model that PERI serves as the primary source of proliferative cells during tendon remodeling, while the TP remains metabolically stable unless affected by environmental stimuli [13, 20, 21].

Inflammatory stimulation with IL‐1β revealed niche‐specific differences in tenocyte plasticity and repair capacity. In TP constructs, IL‐1β strongly suppressed key tenogenic transcription factor *SCX*, consistent with the inflammatory repression of an organized, mature tendon phenotype, and was not rescued by H‐Pro‐Hyp‐OH treatment [25, 26]. *SCX* expression is crucial in the tenogenic cascade, and its downregulation is associated with tenocyte phenotypic deviation [13, 28]. *SCX*‐lineage cells are required for functional tendon healing, since scarless neonatal healing relies on recruitment of *SCX*‐lineage extrinsic (PERI) cells to the injury site [13, 49].

Suppression of the key tenogenic pathway was followed by a significant sustained reduction in total biochemical collagen content, indicating that 500 μg/mL Pro‐Hyp supplementation was insufficient to overcome IL‐1β induced inhibition of matrix synthesis in TP constructs. SHG imaging revealed a significant reduction in angular dispersion in IL‐1β‐treated TP constructs, suggesting a stress‐induced matrix metalloproteinase (MMP) mediated catabolic remodeling rather than active, organized matrix regeneration [23, 24, 25, 26, 27].

In contrast, PERI constructs displayed a strong proliferative and remodeling response to IL‐1β challenge, characterized by significant upregulation of tenogenic/structural markers *COL1A1*, *LOX, DCN*, as well as *CASP3*, consistent with elevated apoptotic signaling. These results are indicative of an activated early repair state compensating for elevated cell turnover with increased matrix production [13]. Under inflammatory conditions, H‐Pro‐Hyp‐OH supplementation effectively restored percent collagen area and increased anisotropy, indicating improved collagen organization despite adverse inflammation response transcriptional signaling.

The differing responses in both cell populations support a post‐injury model in which PERI cells play a responsive, reparative role bolstered by dipeptide treatment [20, 21]. While TP cells show limited regenerative rescue under inflammatory stress, PERI cells exhibit the plasticity required to respond to stressful conditions.

Dexamethasone treatment promoted a distinct transcriptional response in both tendon cell niches, with significant upregulation of *COL1A1*, *LOX*, and *DCN*, indicating activation of matrix synthesis genes despite the known inhibitory effects of steroids on collagen synthesis and tenocyte proliferation in tendon healing [28, 29, 30, 31, 32]. Expression remained upregulated even after dipeptide treatment but was not statistically different from DEX groups.

Relative to PERI constructs, there was a DEX‐induced upregulation of *CSPG4* in TP constructs which could be indicative of a shift from a stable, mature tendon phenotype toward a maladaptive repair state, consistent with findings that glucocorticoid stress drives phenotypic deviation in tenocytes [20, 28, 32]. *SCX* expression remained elevated in DEX‐stressed PERI constructs even after dipeptide treatment, suggesting a sustained tenogenic repair response despite adverse stimuli to potentially reinforce cell differentiation back to a tenogenic phenotype in cells subjected to glucocorticoid stress. Sustained expression of *SCX* and other tenogenic markers in the first responder PERI niche suggests H‐Pro‐Hyp‐OH can promote functional, rather than fibrotic repair in DEX‐stress environments.

The sustained transcriptional activity may be a state of dysregulated or compensatory anabolic repair, contrasting with IL‐1β‐induced inflammatory suppression. Although H‐Pro‐Hyp‐OH supplementation did not restore the DEX‐induced transcriptomic shift in either cell population, it produced a compelling structural rescue in both TP and PERI constructs, emphasizing the disconnect between high mRNA expression of matrix assembly genes and actual collagen deposition. In TP constructs, total collagen content (hydroxyproline) was lower following DEX stressor and was not restored by dipeptide treatment.

H‐Pro‐Hyp‐OH treatment successfully restored percent collagen area and anisotropy in both TP and PERI constructs, indicating improved collagen organization and alignment. Under inflammatory and glucocorticoid stress, increases in SHG‐derived percent collagen area and anisotropy despite lower hydroxyproline levels suggest that H‐Pro‐Hyp‐OH promotes organized fibrillogenesis rather than bulk protein accumulation seen in unstressed controls. This pattern supports a dipeptide‐mediated shift toward matrix organization, in which tenocytes prioritize assembly of aligned fibrillar collagen over total collagen deposition, a critical feature of functional tendon repair to limit scar tissue formation. This is consistent with findings of H‐Pro‐Hyp‐OH driven matrix remodeling and reorganization through β1‐integrin activation [36]. By stimulating lamellipodia‐driven motility and increasing the directional persistence of tenocytes, H‐Pro‐Hyp‐OH may enhance their ability to pull collagen into more aligned, anisotropic structures, even under conditions that suppress overall protein synthesis [36].

The structural rescue observed with H‐Pro‐Hyp‐OH treatment acts on collagen alignment and organization with no corresponding increase in total volume of collagen protein. The mismatch between robust *COL1A1* transcription and low collagen protein levels in stressed constructs suggests that while tenocytes remain transcriptionally active, later stages of the collagen maturation, secretion, or degradation pathways are disrupted [17, 18, 26, 43, 50, 51, 52, 53, 54]. Consequently, while transcriptional activity is upregulated as a repair response, the stress environment compromises the process of protein production, resulting in low collagen accumulation despite high transcriptional activity.

This study had several limitations. We chose two concentrations of H‐Pro‐Hyp‐OH based upon a previous study [36]; however, we have not increased concentrations above 500 μg/mL. It is possible that increased concentrations of dipeptide could have even more beneficial outcomes for the equine TP and PERI cells in 3D constructs. Follow‐up studies are necessary to assess metabolic changes in stressed tenocytes treated with H‐Pro‐Hyp‐OH to determine whether the dipeptide mitigates stress‐induced metabolic constraints on repair capacity, since inflammatory and glucocorticoid stimuli are known to induce metabolic reprogramming where ATP is diverted from costly collagen synthesis [25, 28, 32, 37, 55, 56]. Although H‐Pro‐Hyp‐OH supplementation improved collagen organization, it did not reverse stress‐induced transcriptional shifts, and bulk collagen content remained low, suggesting that H‐Pro‐Hyp‐OH alone is insufficient to completely overcome limitations imposed by inflammatory or glucocorticoid stress. Future studies should evaluate concurrent treatment of H‐Pro‐Hyp‐OH and other therapies to promote a more hospitable environment for active matrix synthesis, in addition to fibril alignment. Importantly, the static in vitro 3D culture system fails to fully capture the complexity of tendon development under physiologically relevant conditions. Mechanical cues are key regulators of the TGF‐β signaling pathway, tenocyte alignment, collagen fibril cross‐linking, and matrix deposition; the lack of these biomechanical inputs in static culture may limit the regenerative efficacy of H‐Pro‐Hyp‐OH [7, 57]. Future work should incorporate TP and PERI coculture or transition to in vivo models to evaluate TP tendon regeneration in the presence of trophic signaling from PERI when bolstered by H‐Pro‐Hyp‐OH treatment.

## 5 Conclusion

Ultimately, this study identifies collagen‐derived dipeptide H‐Pro‐Hyp‐OH as a modulator of tendon repair that enhances matrix organization rather than bulk collagen accumulation in simulated post‐injury environments. Our findings highlight the extrinsic PERI niche as a particularly promising therapeutic target since these cells exhibit superior repair plasticity and more robustly respond with dipeptide‐induced collagen alignment and organization. Although environmental stressors such as inflammation and glucocorticoid stress can overwhelm cellular homeostasis, the ability of H‐Pro‐Hyp‐OH to improve fibril alignment suggests a potential strategy for reducing fibrotic scar formation while promoting functional ECM architecture. These results support further investigation into treatments that leverage the extrinsic niche to drive the assembly of a collagen‐rich, anisotropic matrix during post‐injury equine SDFT repair.

## Author Contributions

Conceptualization and research design: Mienaltowski M; funding acquisition: Mienaltowski M; methodology: Mienaltowski M, Wong A; data acquisition: Wong A, Mienaltowski M; data analysis and interpretation: Wong A, Mienaltowski M; statistical analysis: Wong A, Mienaltowski M; supervision: Mienaltowski M; writing – original draft preparation: Wong A, Mienaltowski M; writing – review and editing: Mienaltowski M and Wong A. All authors have read and approved the final version of the manuscript.

## Conflicts of Interest

The authors declare no conflicts of interest.

## Supporting information


**Figure S1:** Representative SHG images of TP and PERI constructs treated with H‐Pro‐Hyp‐OH. **Figure S2:** Representative SHG images of TP and PERI constructs challenged with IL‐1β or DEX, supplemented with H‐Pro‐Hyp‐OH.

## Data Availability

The data that support the findings of this study are available from the corresponding author upon reasonable request.
