# Supplementary material for: Prolyl‐Hydroxyproline Promotes Structural Matrix Organization in 3D Equine Tendon Proper and Peritenon Post‐Injury Model
Source: J Orthop Res. 2026 Sep 21;44(9):e70284. doi: 10.1002/jor.70284 (PMC13591663; doi:10.1002/jor.70284)
Supplement: Supplementary file 1 [file JOR-44-0-s001.pdf]

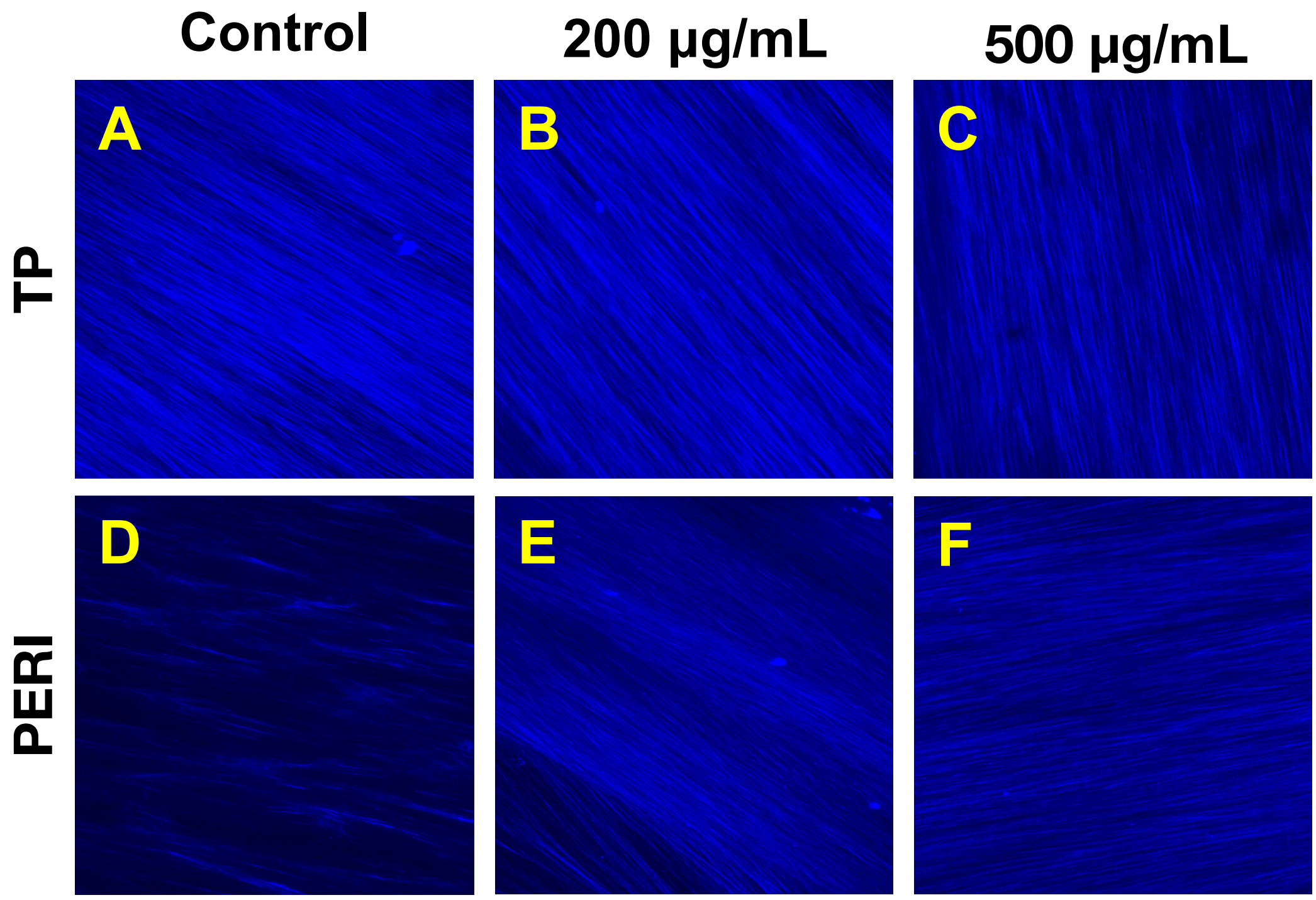

Figure S1. Representative SHG images of TP and PERI constructs treated with H-Pro-Hyp-OH. Images of TP constructs are shown after H-Pro-Hyp-OH treatments of 0 µg/mL **(A)**, 200 µg/mL **(B)**, or 500 µg/mL **(C)**. Images of PERI constructs are shown after H-Pro-Hyp-OH treatments of 0 µg/mL **(D)**, 200 µg/mL **(E)**, or 500 µg/mL **(F)**.

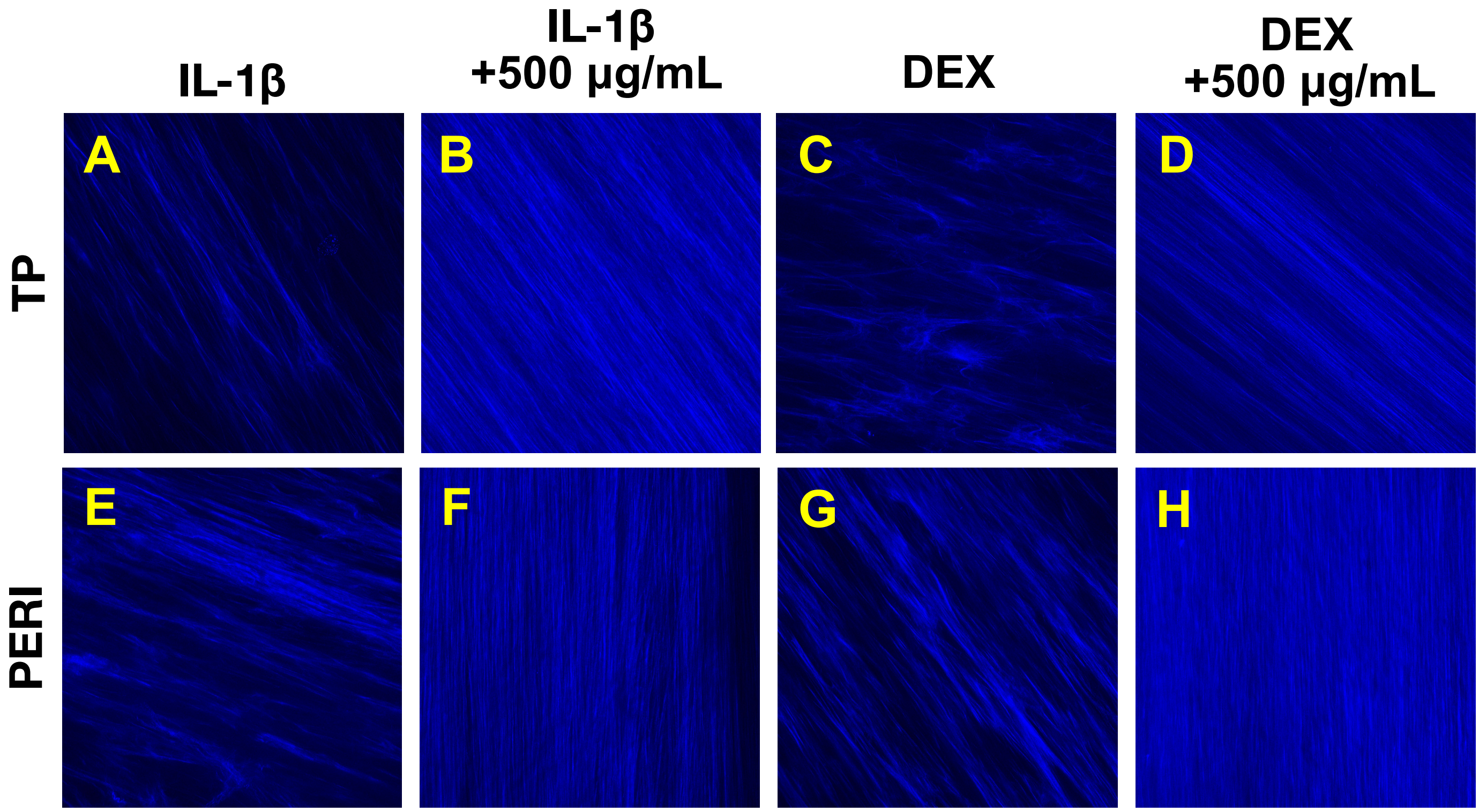

Figure S2. Representative SHG images of TP and PERI constructs challenged with IL-1β or DEX, supplemented with H-Pro-Hyp-OH. Images of TP constructs are shown after challenge with IL-1β **(A)**, IL-1β+500 µg/mL H-Pro-Hyp-OH **(B)**, DEX **(C)**, or DEX+500 µg/mL H-Pro-Hyp-OH **(D)**. Images of PERI constructs are shown after challenge with IL-1β **(E)**, IL-1β+500 µg/mL H-Pro-Hyp-OH **(F)**, DEX **(G)**, or DEX+500 µg/mL H-Pro-Hyp-OH **(H)**.

Overall, minimal changes in fiber characteristics were observed in TP dipeptide controls **(Fig. S1 A-C)**. However, obvious visual improvements were seen following dipeptide treatment in PERI controls **(Fig. S1 D-F)**. Likewise, visual changes are seen in both stressed TP constructs **(Fig. S2 A-D)** and stressed PERI groups **(Fig. S2 E-H)** with similar changes in SHG signal intensity, fiber density, and packing. Collectively, improvements seen with dipeptide supplementation suggest that its addition promotes collagen matrix organization across conditions, with evidence of structural rescue under stress and greater baseline responsiveness in PERI constructs.
